# Investigating the relationship between malaria incidence and public health infrastructure in sub-Saharan Africa

**DOI:** 10.1186/s12936-025-05751-6

**Published:** 2025-12-26

**Authors:** Jaebin Shin

**Affiliations:** Stanford Online High School, 415 Broadway, Redwood City, CA 94063 USA

**Keywords:** Malaria, Malaria incidence, Water access, Sanitation access, Machine learning, Infrastructure, Health disparities, Environmental determinants of health, Predictive modeling

## Abstract

**Background:**

Malaria remains a major public health challenge in sub-Saharan Africa, and its burden may be influenced by access to clean water, sanitation, and childhood vitamin A supplementation. Understanding how these indicators relate to malaria incidence can help inform targeted prevention strategies.

**Methods:**

Country-level data from global health databases were analyzed using nonparametric statistical tests and machine learning models. The Kruskal–Wallis test and Dunn’s post hoc comparisons were used to assess differences in malaria incidence across categories of water and sanitation access. Cliff’s delta was used to measure effect sizes. Tree-based machine learning models and logistic regression were trained to evaluate the predictive strength of the three indicators.

**Results:**

Significant differences in malaria incidence were found across water and sanitation access groups, with the lowest access groups consistently exhibiting the highest incidence. Cliff’s delta indicated large effect sizes, particularly between low and high access categories. Vitamin A supplementation showed statistically significant group differences, though effect sizes were generally small. Tree-based machine learning models showed moderate predictive performance and outperformed logistic regression in classification accuracy and recall.

**Conclusions:**

Access to clean water and adequate sanitation are strongly associated with lower malaria incidence, underscoring their importance in malaria control efforts. While vitamin A supplementation shows weaker associations, it may still interact with broader health conditions. These findings highlight the essential role of basic infrastructure in reducing malaria burden and demonstrate the potential of predictive modeling to support future global health research.

## Background

Malaria is one of the most severe infectious diseases globally, particularly among vulnerable populations in sub-Saharan Africa. In 2022, malaria caused an estimated 610,000 deaths out of 249 million cases worldwide, with 95% of these deaths occurring in Africa [1]. Malaria disproportionately affects children under five and pregnant women and is one of the leading causes of infant and maternal mortality in areas with limited access to health care.

In addition to its immediate health impacts, such as fever, chills, anemia, and potentially fatal cerebral complications, malaria has far-reaching socioeconomic consequences, especially in impoverished populations with inadequate infrastructure and health services. Studies have established a strong link between malaria incidence and intrinsic environmental and public health factors, including poor sanitation, insufficient clean water supply, and inadequate nutrition [2]. For instance, a lack of access to clean water not only affects hydration and general well-being but also encourages mosquito breeding grounds, contributing directly to disease transmission. Clean water, therefore, serves as both a preventive measure and a determinant of broader community health.

Healthcare infrastructure plays a similarly pivotal role. Access to timely diagnosis, treatment, and prevention tools has been shown to significantly reduce malaria morbidity and mortality. Areas with limited availability of healthcare services, whether due to geographic, financial, or systemic barriers, often experience delayed treatment and misdiagnosis, allowing the disease to progress unchecked. Healthcare service coverage, including community-level interventions and routine care, has been consistently linked to improved disease management and outcomes [3].

Nutrition, too, has been identified as a critical yet often underemphasized factor influencing malaria outcomes, alongside water and sanitation. Vitamin A deficiency, common in malaria-endemic regions, compromises immune function and heightens vulnerability to infection. Clinical studies have shown that vitamin A supplementation can reduce malaria severity, particularly among children [4]. This suggests that nutritional status is not only a background condition but an active modifier of disease progression and recovery.

Despite existing measures such as bed nets, antimalarial drugs, and vaccination, malaria continues to thrive in populations facing persistent environmental and social challenges. Understanding how clean water access, healthcare availability, and vitamin A coverage interact with malaria outcomes is essential for designing more effective and equitable interventions.

This study investigates the correlation between malaria incidence and three key factors: access to clean water, access to healthcare services, and coverage of vitamin A supplementation. Using data analysis and statistical tests, the research will assess the strength and significance of these associations. In addition, machine learning models will be applied to estimate the likelihood of malaria incidence under different conditions, offering predictive insights that can inform targeted public health strategies and future interventions.

## Methods

### Key variables

The data used in this research originates from surveys conducted by multiple organizations, with the main sources traceable through the World Bank database [5]. Along with malaria incidence, three key predictors were identified and will serve as the primary variables in the analysis.

#### Malaria incidence

Malaria incidence data were originally available from the World Health Organization’s World Malaria Report, the Global Health Observatory Data Repository, and related World Bank repositories [6], covering the period from 2000–2022. However, due to missing data in earlier years, the final analysis was limited to complete records from the 2014–2022 period. Malaria incidence refers to the number of new confirmed malaria cases per 1,000 individuals in the population at risk.

#### Water supply predictors

Two variables were used to represent water supply access: “safely managed” water supply and “least basic” water supply. “Safely managed” water supply refers to drinking water from an improved source that is accessible on premises, available when needed, and free from fecal and priority chemical contamination. Examples include piped water, boreholes or tubewells, protected dug wells, protected springs, and packaged or delivered water. The “least basic” category also includes sources with collection times of no more than 30 min round-trip, focusing on accessibility.

The datasets for both the “safely managed” and “least basic” water supply (urban and rural) were obtained from the WHO/UNICEF Joint Monitoring Programme (JMP) for Water Supply, Sanitation and Hygiene [7]. These data originally spanned 2000–2022, but due to incomplete data in earlier years, the analysis focused on 2014–2022, where complete data were available.

#### Sanitation services predictors

Two variables were used to represent access to sanitation services: “safely managed” sanitation services and “least basic” sanitation services. “Safely managed” sanitation services include improved facilities not shared with other households and where excreta are safely disposed of or treated off-site. The “least basic” category also includes “basic” services (improved facilities not shared with other households but without requiring safe disposal of excreta).

The datasets for both “safely managed” and “least basic” sanitation services (urban and rural) were also sourced from the WHO/UNICEF JMP [7]. These data were originally collected over 2000–2022, but the analysis was based on the 2014–2022 subset with complete entries.

#### Vitamin A supplementation

Vitamin A supplementation coverage refers to the percentage of children aged 6–59 months who received two high-dose vitamin A supplements within a calendar year. Data from UNICEF global databases [8] originally covered 2000–2022, but only complete records from 2014–2022 were included in this analysis. This indicator measures the reach of national supplementation programs aimed at reducing child vulnerability to infectious diseases, including malaria.

### Data cleaning and preparation

The initial dataset included a comprehensive table of malaria incidence and predictor variables for sub-Saharan African countries over the 2014–2022 period. However, data availability varied inconsistently across years and countries, and some countries lacked data in earlier years, while others had missing values in later periods. To ensure consistency and reliability of the analysis, 144 rows were removed due to missing or incomplete entries. 342 rows with complete records across all variables for all target years were retained.

To enable structured statistical analysis and machine learning modeling, continuous variables were categorized into four levels based on interquartile range (IQR) thresholds. These four categories are:

- **Low** (below 25th percentile)
- **Moderate-Low** (25th–50th percentile)
- **Moderate-High** (50th–75th percentile)
- **High** (above 75th percentile)

This IQR-based categorization was chosen to account for the non-normal distribution of the data and to ensure a balanced representation of observations within each category, reflecting the natural quartiles of the dataset. This approach was preferred over methods like equal-width binning or fixed thresholds because it aligns with the actual spread and skewness of the data.

The IQR-based thresholds for each key variable are as shown on Table 1.

**Table 1:** IQR thresholds used for categorizing key variables

Variable	25th percentile	Median (50th)	75th percentile
Malaria Incidence (per 1,000)	43.83	185.42	294.71
Least Basic Water Access (%)	57.99	68.40	82.91
Least Basic Sanitation (%)	20.74	36.84	60.04
Vitamin A Supplementation Coverage (%)	26.00	57.00	86.75

To describe regional patterns, countries were grouped by subregion (Northern Africa, Western Africa, Eastern Africa, Central Africa, Southern Africa) using the United Nations geoscheme. This regional classification was shown on Table 2 to improve the interpretability of results and highlight differences between areas.

**Table 2:** Demographics of all available years in each category per included country

	All Country-Years (n* = 342)	Northern Africa (n = 7)	Western Africa (n = 127)	Eastern Africa (n = 85)	Central Africa (n = 54)	Southern Africa (n = 69)
Malaria incidence						
Rare	55 (16%)	2 (29%)	3 (2%)	12 (14%)	8 (15%)	30 (43%)
Moderate low	99 (29%)	5 (71%)	34 (27%)	45 (53%)	0 (0%)	15 (22%)
Moderate high	91 (27%)	0 (0%)	21 (17%)	23 (27%)	32 (59%)	15 (22%)
High	97 (28%)	0 (0%)	69 (54%)	5 (6%)	14 (26%)	9 (13%)
Least basic water						
Low	107 (31%)	2 (29%)	19 (15%)	50 (59%)	24 (44%)	12 (17%)
Moderate low	102 (30%)	5 (71%)	38 (30%)	26 (31%)	7 (13%)	26 (38%)
Moderate high	92 (27%)	0 (0%)	55 (43%)	9 (11%)	18 (33%)	10 (14%)
High	41 (12%)	0 (0%)	15 (12%)	0 (0%)	5 (9%)	21 (30%)
Least basic sanitation services						
Low	106 (31%)	0 (0%)	42 (33%)	33 (39%)	31 (57%)	0 (0%)
Moderate low	107 (31%)	6 (86%)	42 (33%)	30 (35%)	0 (0%)	29 (42%)
Moderate high	97 (28%)	1 (14%)	42 (33%)	14 (17%)	22 (41%)	18 (26%)
High	32 (9%)	0 (0%)	1 (1%)	8 (9%)	1 (2%)	22 (32%)
Vitamin A supplementation						
Low	78 (23%)	2 (29%)	26 (21%)	18 (21%)	20 (37%)	12 (17%)
Moderate low	88 (26%)	2 (29%)	26 (21%)	18 (21%)	13 (24%)	29 (42%)
Moderate high	86 (25%)	2 (29%)	31 (24%)	27 (32%)	9 (17%)	17 (25%)
High	90 (26%)	1 (14%)	44 (35%)	22 (26%)	12 (22%)	11 (16%)

^*^n = 342 refers to the total number of country-year observations analyzed after data cleaning, covering nine years of data for each included country

Notably, for malaria incidence, the category labeled “Rare” in Table 2 corresponds to the “Low” group defined here (below the 25th percentile). The term “Rare” was used in the demographic breakdown table to emphasize the practical meaning of very low incidence, but methodologically, it follows the same IQR-based grouping.

These categorical groupings were then applied uniformly in subsequent statistical tests and machine learning models to allow consistent comparison across approaches.

## Results

### Normality testing

Before conducting statistical analyses, normality tests were performed to determine whether the data conformed to a normal distribution, an important assumption for choosing between parametric and nonparametric methods. The D’Agostino-Pearson omnibus test was applied to all variables.

Results indicated significant deviations from normality across all variables (all *p* < 0.001). For example, malaria incidence showed a test statistic of 86.44 (*p* ≈ 1.7 × 10⁻^19^), and vitamin A supplementation had a markedly non-normal distribution with a statistic of 911.31 (*p* < 1 × 10⁻^198^).

Given these findings, nonparametric methods were selected for subsequent analyses: Spearman’s rank correlation for assessing relationships between variables, and the Kruskal–Wallis test with Dunn’s post hoc comparisons for examining group differences based on IQR-based categorical groupings.

### Correlation analysis

**Fig. 1:**
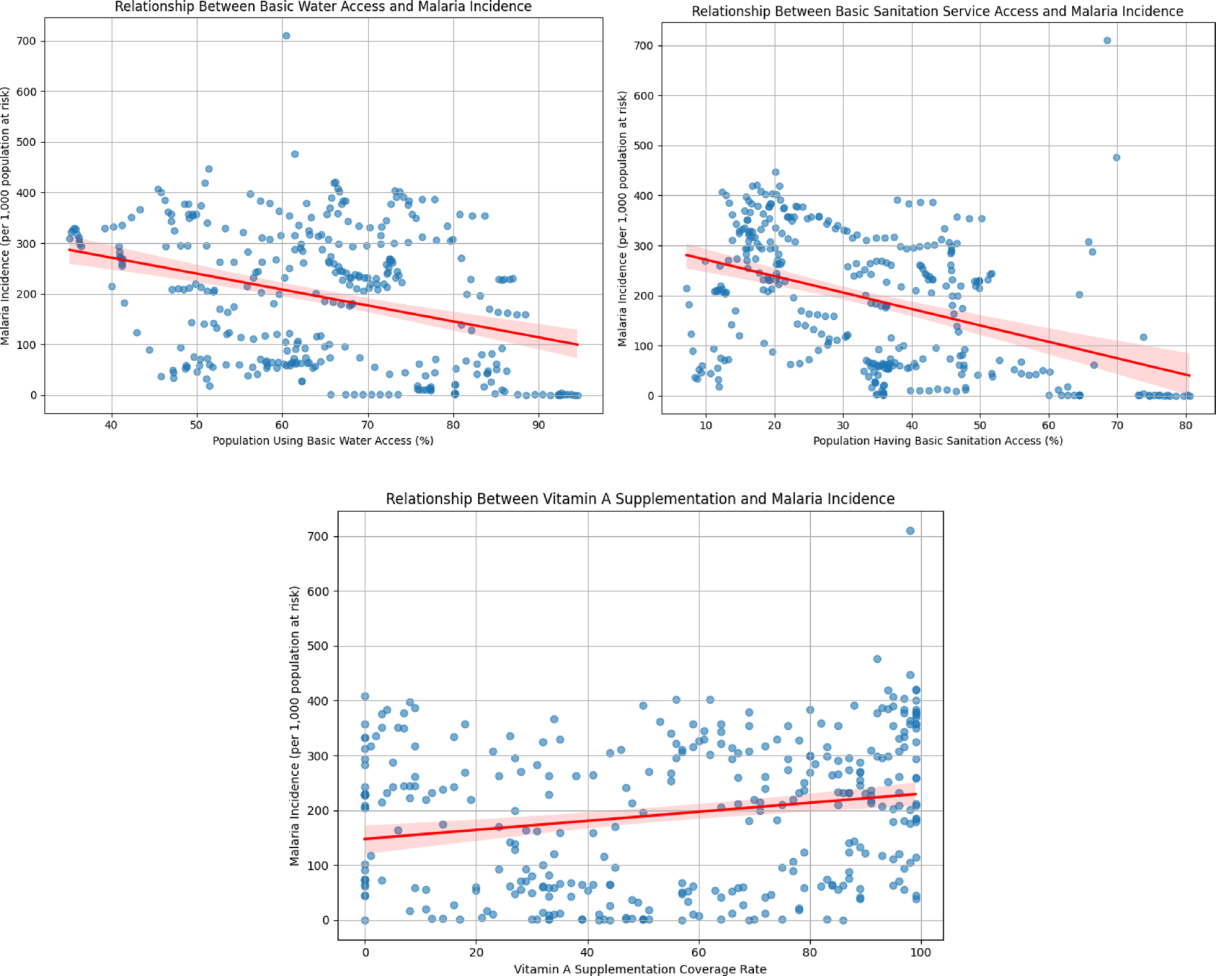
Correlations between malaria incidence and independent variables. X-axis represents each independent variable, and Y-axis shows malaria incidence. The red line indicates the line of best fit, and the shaded area marks the error bounds. Left: Water access vs. malaria incidence. Middle: Vitamin A supplementation versus malaria incidence. Right: Sanitation access vs. malaria incidence

Due to non-normality in the data, Spearman’s rank correlation was used to examine the relationship between malaria incidence and each predictor variable. This nonparametric method is well suited for data that do not meet the assumptions required for traditional Pearson correlation, as it measures monotonic relationships without assuming normal distribution (Fig. 1).

A moderate negative correlation was found between malaria incidence and access to sanitation services (ρ = – 0.423, *p* < 0.001), indicating that higher levels of basic sanitation are associated with lower malaria incidence. A weaker but still significant negative correlation was observed with access to clean water (ρ = – 0.321, *p* < 0.001). In contrast, vitamin A supplementation showed a positive but weak correlation with malaria incidence (ρ = 0.224, *p* < 0.001), which may reflect a more complex or confounded relationship. For example, countries with higher malaria burden may be more likely to implement broader vitamin A supplementation programs.

### Group differences in malaria incidence by service categories

While correlation and regression analyses identified overall associations between malaria incidence and continuous measures of water access, sanitation services, and vitamin A supplementation, they do not reveal how malaria incidence may differ across different levels of service coverage. To explore these potential group-wise disparities, the Kruskal–Wallis test was used to detect overall differences in incidence between groups (categorized previously in the methods), followed by Dunn’s post hoc comparisons to identify specific group contrasts. Additionally, Cliff’s delta was used to quantify the magnitude and direction of these differences, offering a more detailed understanding of how service access levels relate to malaria burden.

#### Water supply

A Kruskal–Wallis test showed a significant difference in malaria incidence across water supply categories (H = 47.364, ρ < 0.001), indicating that incidence levels are not equally distributed across groups.

**Fig. 2:**
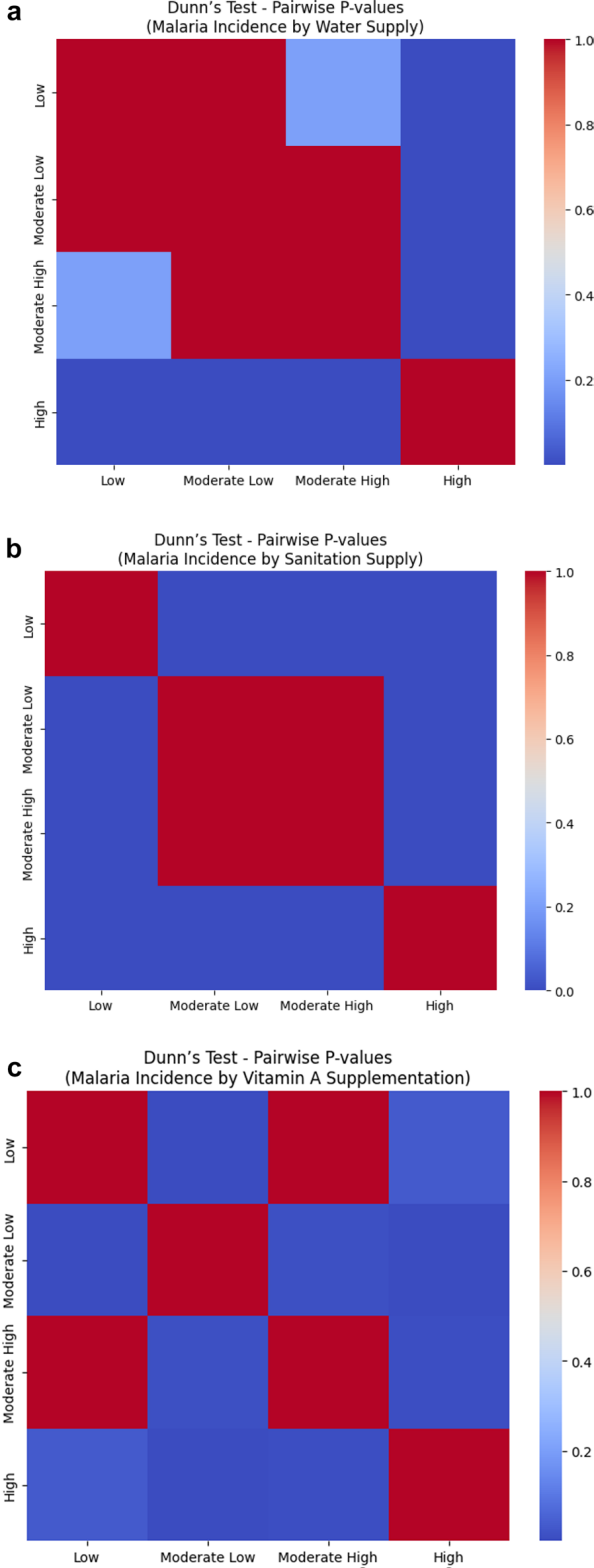
**a** Dunn’s post hoc test heat map for malaria incidence across water supply categories. Each cell shows the adjusted *p*-value for pairwise comparisons. Darker blue indicates a lower *p*-value (stronger statistical significance), while red indicates a *p*-value closer to 1 (not significant). **b** Dunn’s post hoc test heat map for malaria incidence across sanitation supply categories. Each cell shows the adjusted *p*-value for pairwise comparisons. Darker blue indicates a lower *p*-value (stronger statistical significance), while red indicates a *p*-value closer to 1 (not significant).** c** Dunn’s post hoc test heat map for malaria incidence across vitamin A supplementation categories. Each cell shows the adjusted *p*-value for pairwise comparisons. Darker blue indicates a lower p-value (stronger statistical significance), while red indicates a *p*-value closer to 1 (not significant)

Follow-up analysis using Dunn’s test revealed that countries in the High water supply group had significantly different malaria incidence compared to those in the Low, Moderate Low, and Moderate High groups. All other comparisons were not significant (Fig. 2a).

Cliff’s Delta helped quantify the size and direction of these differences. For example, there was a 69.4% chance that a randomly selected country from the Low group would have a higher malaria incidence than one from the High group (δ = 0.694). Similarly, countries in the Moderate Low group are 64.2% more likely to have higher incidence than those in the High group, and the Moderate High vs High comparison also showed a substantial difference (δ = 0.544). These large effects reinforce the practical impact of water access on malaria burden.

#### Sanitation services

Malaria incidence also varied significantly across sanitation service levels (H = 71.969, ρ < 0.001). Dunn’s test showed that the High sanitation group had significantly lower malaria incidence compared to the Low, Moderate Low, and Moderate High groups. The only non-significant difference was between Moderate Low and Moderate High, suggesting similar malaria levels between those two (Fig. 2b).

Cliff’s Delta results showed that there is a 77.5% chance that a country in the Low sanitation group has higher malaria incidence than one in the High group (δ = 0.775, large effect). Comparisons between Moderate groups and High were also meaningful, with countries in the Moderate Low and Moderate High groups being roughly 68–67% more likely to have higher malaria incidence than those in the High group. Even the differences between Low and Moderate groups were noticeable, though smaller in magnitude (δ ≈ 0.37–0.41, medium effect).

#### Vitamin A supplementation

A Kruskal–Wallis test also revealed significant differences in malaria incidence across vitamin A supplementation levels (H = 42.312, ρ < 0.001). Dunn’s test showed that most pairwise comparisons were significant, except for the Low vs. Moderate High group, where incidence levels were statistically similar (Fig. 2c).

While several comparisons were statistically significant, most of the Cliff’s Delta values showed only small effect sizes, meaning that the actual magnitude of difference was limited. For example, there was a 33% chance that a country in the Low group would have a higher incidence than one in the Moderate Low group (δ = 0.330, small), and a – 25.9% difference between Low and High, indicating slightly lower incidence in the High group. The only large effect appeared between Moderate Low and High (δ = – 0.557), showing a more meaningful gap in malaria burden, favoring countries with higher supplementation.

### Machine learning prediction models

To model malaria incidence levels, multivariate classification models were trained using three predictor variables. These multivariate models allow us to evaluate the contribution of each factor while accounting for the others, capturing the joint influence of multiple social determinants on malaria incidence. The model prediction results are shown on Table 3.

**Table 3:** Model prediction results

	Accuracy	Precision	Recall	F1 score	Cohen’s Kappa score
Logistic regression	0.29	0.20	0.31	0.24	0.067
Decision tree	0.41	0.42	0.40	0.39	0.19
Random forest	0.38	0.39	0.39	0.38	0.16

Accuracy measures the overall proportion of correct predictions. Precision shows how many of the predicted positive cases were actually correct, while Recall reflects how many of the actual positive cases were successfully identified. The F1 Score balances precision and recall into a single metric. Cohen’s Kappa Score measures how much better the model performed compared to random guessing, adjusting for chance agreement.

**Fig. 3:**
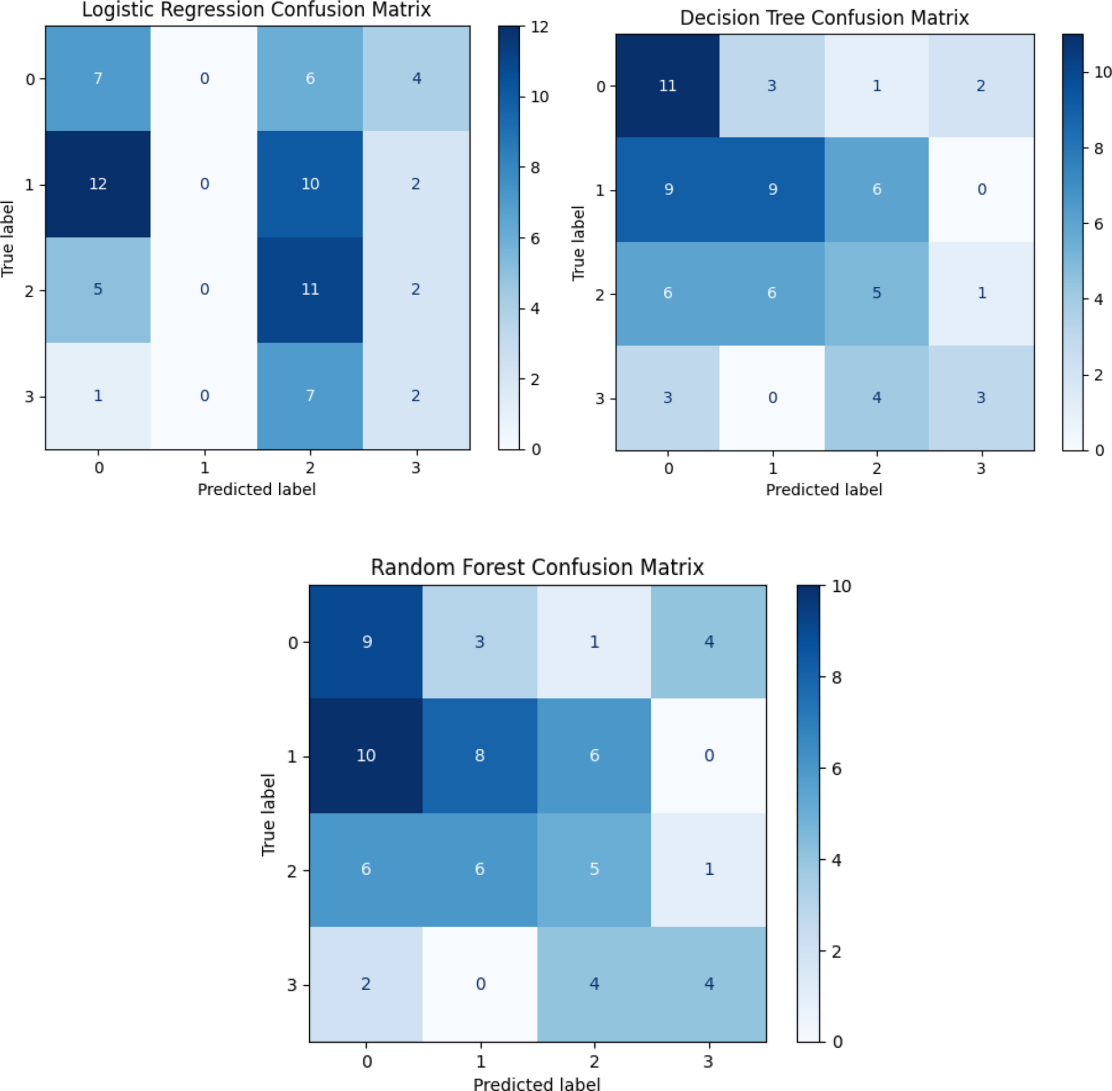
Confusion matrices for each model predicting malaria incidence categories. True values are on the y-axis, predicted values on the x-axis. Darker cells indicate higher counts. Top Left: Confusion matrix for Logistic Regression. Top Right: Confusion matrix for Decision Tree. Bottom: Confusion matrix for Random Forest. *Note:* Class 0 corresponds to the Low malaria incidence category, Class 1 to Moderate Low, Class 2 to Moderate High, and Class 3 to High incidence

Three classification models were trained to predict malaria incidence categories: Logistic Regression, Decision Tree, and Random Forest. Among these, the Decision Tree achieved the highest performance with an accuracy of 41%, a macro-averaged precision of 0.42, a recall of 0.40, and an F1 score of 0.39. Random Forest performed slightly below, with an accuracy of 38%, a precision of 0.39, a recall of 0.39, and an F1 score of 0.38. Logistic Regression showed the weakest performance, with an accuracy of just 29%, a precision of 0.20, and a low F1 score of 0.24. Cohen’s Kappa values reinforced these trends, with the Decision Tree scoring the highest agreement beyond chance (κ = 0.19), followed by Random Forest (κ = 0.16), and Logistic Regression (κ = 0.07), indicating poor performance and weak agreement with the true class distribution (Fig. 3).

Analysis of the confusion matrices further revealed key differences in each model’s behavior. Logistic Regression consistently misclassified observations, failing to correctly predict any instances of Class 1 and heavily mislabeling Class 3 cases as Class 2. The Decision Tree improved on this, correctly classifying 11 instances of Class 0 and 9 instances of Class 1, though it still showed overlap between Class 1 and neighboring classes, such as Class 0 and 2. Random Forest displayed similar patterns, with 9 correct predictions for Class 0 and 8 for Class 1, while also capturing 4 correct predictions of Class 3. However, it showed slightly more confusion between Class 3 and Class 2 than the Decision Tree. Overall, the tree-based models demonstrated clearer class separation and more balanced classification across all categories compared to Logistic Regression.

## Discussion

This analysis examined the association between access to clean water, sanitation services, and vitamin A supplementation and malaria incidence across sub-Saharan African countries. Consistent results showed that low access to water and sanitation is associated with significantly increased malaria incidence, supported by both group difference tests and effect size estimates. For example, countries in the lowest groups for water and sanitation were over 69% and 77% more likely, respectively, to have higher malaria incidence than those in the highest groups, showing strong and meaningful differences. Even partial improvements from low to moderate service levels showed statistically significant differences in burden. Vitamin A supplementation showed more inconsistent results: while there were statistically significant differences, they tended to be small in practice, suggesting that vitamin A coverage may reflect national health program targeting rather than being a direct driver of malaria incidence. These findings align with broader global health literature highlighting the importance of improving basic infrastructure, particularly water and sanitation, as part of disease control strategies rather than solely development goals.

Among the machine learning models tested, tree-based models such as the Decision Tree and Random Forest outperformed logistic regression on nearly all evaluation measures. These models were more accurate, recalled more cases correctly, and showed higher agreement with true labels, suggesting their effectiveness in capturing nonlinear relationships and threshold effects common in public health data. While overall predictive accuracy remained limited, including these models added an applied perspective and demonstrated the potential of more flexible, interpretable tools in helping identify patterns and guide targeted interventions in global health.

The analysis also had several strengths. It used robust nonparametric methods suited for skewed data, a large dataset covering multiple countries, and complemented statistical significance testing with effect size estimation (Cliff’s Delta), adding depth to understanding real-world differences. Testing machine learning models further added an applied dimension to the study. However, there were key limitations. Converting continuous variables (like percentage of water access) into categorical groups using IQR-based binning simplified comparisons, but likely reduced the precision of both statistical tests and predictive models. Additionally, the modest accuracy of the machine learning models reflects the complexity of predicting health outcomes from a limited number of discretized predictors.

In conclusion, this analysis highlights the essential role of basic services and infrastructure in shaping malaria outcomes. The strongest and most consistent finding was the impact of water and sanitation access, where countries with better service levels showed much lower malaria incidence. Future studies could refine these insights by using continuous predictors directly, accounting for regional or temporal patterns, and including additional factors like mosquito control, urbanization, or climate data. Further development of mathematical models that incorporate spatial, temporal, and environmental predictors could also improve the understanding and prediction of malaria risk. Strengthening basic services remains a cornerstone of malaria control, and findings like these support scaling interventions in areas with the highest burden and lowest access.

## Data Availability

The datasets generated and/or analysed during the current study are available in the World Bank repository (License: CC BY-4.0): [safely managed water](https:/data.worldbank.org/indicator/SH.H2O.SMDW.ZS) (ID: SH.H20.SMDW.ZS), [least basic water](https:/data.worldbank.org/indicator/SH.H2O.BASW.ZS) (ID: SH.H20.BASW.ZS), [malaria incidence](https:/data.worldbank.org/indicator/SH.MLR.INCD.P3) (ID: SH.MLR.INCD.P3), [safely managed sanitation](https:/data360.worldbank.org/en/indicator/WB_WDI_SH_STA_SMSS_ZS) (ID: WB\_WDI\_STA\_SMSS\_ZS), [least basic sanitation](https:/data360.worldbank.org/en/indicator/WB_WDI_SH_STA_BASS_ZS) (ID: WB\_WDI\_SH\_STA\_BASS\_ZS), and [vitamin A](https:/data360.worldbank.org/en/indicator/WB_WDI_SN_ITK_VITA_ZS) (ID: WB\_WDI\_SN\_ITK\_ZS).

## Code availability

The code used in this study is available on Kaggle at https://www.kaggle.com/code/jaebins/malaria-research.
